# SCDRHA: A scRNA-Seq Data Dimensionality Reduction Algorithm Based on Hierarchical Autoencoder

**DOI:** 10.3389/fgene.2021.733906

**Published:** 2021-08-27

**Authors:** Jianping Zhao, Na Wang, Haiyun Wang, Chunhou Zheng, Yansen Su

**Affiliations:** ^1^College of Mathematics and System Sciences, Xinjiang University, Ürümqi, China; ^2^Key Lab of Intelligent Computing and Signal Processing of Ministry of Education, School of Artificial Intelligence, Anhui University, Hefei, China

**Keywords:** scRNA-seq, dimensionality reduction, graph autoencoder, graph attention networks, noise reduction

## Abstract

Dimensionality reduction of high-dimensional data is crucial for single-cell RNA sequencing (scRNA-seq) visualization and clustering. One prominent challenge in scRNA-seq studies comes from the dropout events, which lead to zero-inflated data. To address this issue, in this paper, we propose a scRNA-seq data dimensionality reduction algorithm based on a hierarchical autoencoder, termed SCDRHA. The proposed SCDRHA consists of two core modules, where the first module is a deep count autoencoder (DCA) that is used to denoise data, and the second module is a graph autoencoder that projects the data into a low-dimensional space. Experimental results demonstrate that SCDRHA has better performance than existing state-of-the-art algorithms on dimension reduction and noise reduction in five real scRNA-seq datasets. Besides, SCDRHA can also dramatically improve the performance of data visualization and cell clustering.

## Introduction

With the rapid development of single-cell RNA sequencing (scRNA-seq) technology, the research of transcriptomics has changed dramatically (Tang et al., 2013; Xi et al., 2018, 2020). On the one hand, the cell is the unit of an organism, mining data at the single-cell level can help researchers probe the essence and laws of living activities. On the other hand, the scale of scRNA-seq data obtained by researchers is growing, which brings enormous challenges in analysis and computation (Kiselev et al., 2019; Yu et al., 2021). How to transform a high-dimension data into low-dimension embedding while preserving the topological structure of raw data plays an indispensable role in scRNA-seq analysis. Besides, the high noise in scRNA-seq data will make it far too difficult to reduce dimension. One of the most challenging noises is the dropout events, which caused zero inflation in scRNA-seq data (Zhang and Zhang, 2018). The low RNA capture rate leads to the detection failure of an expressed gene resulting in a “false” zero count observation, which is defined as a dropout event. The zero counts consist of “false” zero counts and “true” zero counts, where the true counts represent the lack of expression of a gene in a specific cell, and the false zero counts are dropout events. A large number of false zero counts will lead to unreliable results of visualization, clustering, and pseudotime inference. Thus, noise reduction is integral for scRNA-seq data analysis as well as dimension reduction.

The new challenges of scRNA-seq data bring new opportunities, these data have spurred the millions of algorithms to derive novel biological insights (Hie et al., 2020; Wang et al., 2021a,b). Because of the high-dimensionality of scRNA-seq, many dimension reduction methods have been proposed for scRNA-seq data. Some of these methods fail to consider zero inflation (dropout) of the scRNA-seq data, including uniform manifold approximation and projection (UMAP) (Becht et al., 2019) and single-cell graph autoencoder (scGAE) (Luo et al., 2021). UMAP is a non-linear dimensionality reduction technique, which is a universal method in high-dimensional gene expression analysis. scGAE is a dimensionality reduction method based on graph autoencoder, which can preserve topological structure in scRNA-seq data. Nevertheless, these methods ignore the impact of dropout events on the output.

On the contrary, many single-cell analysis algorithms take dropout events into account, including zero-inflated factor analysis (ZIFA) (Pierson and Yau, 2015), zero-inflated negative binomial (NB)-based wanted variation Extraction (ZINB-WaVE) (Risso et al., 2018), deep count autoencoder (DCA) (Eraslan et al., 2019), and single-cell model-based deep embedded clustering (scDeepCluster) (Tian et al., 2019). ZIFA focuses on dropout events and assumes the dropout rate for a gene depends on the expression level. However, such a strong assumption lacks flexibility, and it is not quite suitable for real datasets. To solve this challenge, ZINB-WaVE has been proposed, which is general and flexible and uses a zero-inflated negative binomial (ZINB) (Risso et al., 2018) model. Nonetheless, ZIFA and ZINB-WaVE have large computation cost; hence, these methods are not fit for large-scale data. DCA is a deep learning method based on autoencoder in an unsupervised manner, which can be applied to datasets of millions of cells. Different from regular autoencoder, the DCA proposes a ZINB model-based loss function substitute for the conventional mean square error loss function to depict scRNA-seq data better. Based on the framework of DCA, scDeepCluster adds the random Gaussian noise into the encoder to improve the embedded feature representation and executes clustering tasks using deep embedded clustering on latent space. However, both DCA and scDeepCluster are not taking the cell–cell relationships into account.

The recently proposed graph attention network (GAT) (Veličković et al., 2018) is a novel neural network architecture that operates on graph-structured data, which preserves the topological structure in a latent space. In this work, we build the graph autoencoder based on GAT to project the data into a low-dimensional latent expression and maintain the topological structure among cells as possible. Considering the input of the graph autoencoder is single-cell graphs of node matrices and adjacency matrix, the adjacency matrix among cells built by the K-nearest-neighbor (KNN) algorithm is quite considerable for graph autoencoder. Nevertheless, the adjacency matrix will be distorted by the impact of the high sparsity of scRNA-seq data on the KNN algorithm. Therefore, we focus on the impact of dropout events on the output of the KNN algorithm and utilize a scalable denoising method DCA to mitigate zero inflation caused by dropout events. Because the raw data and reconstructed data by DCA have the same dimension, we implement initial dimensionality reduction for the reconstructed data by using principal component analysis (PCA). Based on the latent space constructed by PCA, we build a graph autoencoder to reduce the dimension and get a low dimensional embedding for visualization and clustering. These are the motivations behind our new method SCDRHA. We extensively evaluate our approach with competing methods using five real datasets; the experimental results demonstrate that SCDRHA has better performance than the existing state-of-the-art algorithms on dimension reduction and noise reduction. Besides, SCDRHA can also dramatically improve the performance of data visualization and cell clustering.

## Materials and Methods

**FIGURE 1:**
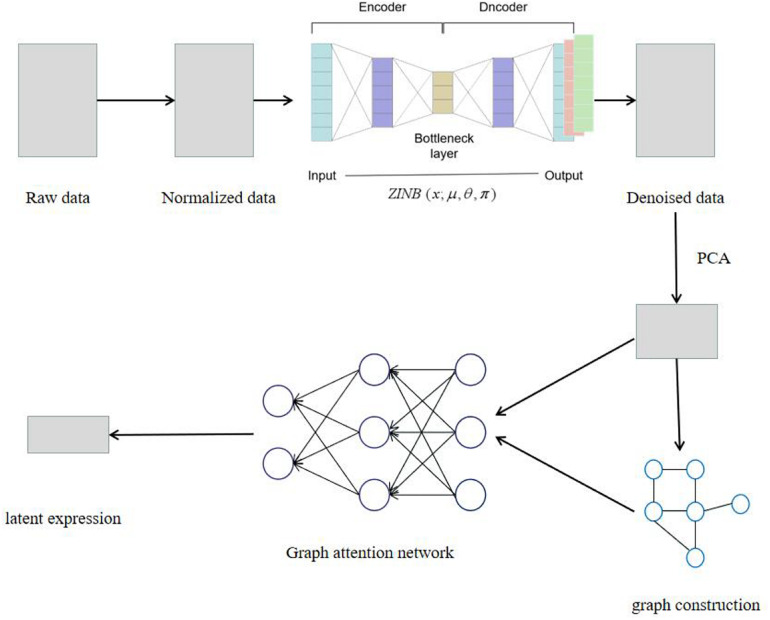
The model architecture of SCDRHA. First, we normalize the raw data. Then, we use deep count autoencoder (DCA) to denoise the data. Ultimately, we use the compressed matrix by PCA and adjacency matrix as the input of graph autoencoder based on graph attention network (GAT) and get a low-dimensional embedding.

The SCDRHA pipeline for scRNA-seq data analysis consists of two core modules (Figure 1). The first model is DCA to alleviate dropout events, which is learned by the ZINB model-based autoencoder. The second model is a graph autoencoder based on GAT, which maps the denoised data by DCA to a low-dimensional latent representation.

### Data Preprocessing

To begin, suppose that we have a raw scRNA-seq count matrix C, which is filtered out genes with no count in any cell. C can be represented as a P-by-N dimensional matrix, where P is defined as the total number of genes, N is defined as the total number of cells, and *c*_*ij*_ represents the expression value of gene *i* in cell *j*.

In this work, we first preprocess the raw scRNA-seq count data, including log transformation and z-score normalization. We have a normalized output X, which is given by

(1) $$X^{\prime }=log_{2}\left(1+diag{\left(s_{j}\right)}^{-1}C\right),$$

(2) $$X=zscore\left(X^{\prime }\right),$$

where *s_j_* is the size factor for every cell *j*. The advantage of data preprocessing is to preserve the impact of library size differences and transform discrete values to become continuous, allowing for greater flexibility for the subsequent modeling.

### Deep Count Autoencoder

To denoise the data after preprocessing and capture the characters of scRNA-seq data, we employ DCA based on the ZINB model, so that we can obtain denoised data, which is beneficial to the stability and accuracy of the subsequent KNN algorithm. Taking the count distribution, overdispersion, and high sparsity of scRNA-seq data into account, DCA applies a ZINB model based on autoencoder to depict the characters of the data, and the loss function of the autoencoder is the likelihood of ZINB distribution.

The ZINB distribution is a mixture model that consists of two components: a point mass at zero and a negative binomial (NB) component.

(3) $$NB\left(x;\mu ,\theta \right)=\frac{\Gamma \left(x+\theta \right)}{\Gamma \left(\theta \right)\Gamma \left(x+1\right)}{\left(\frac{\theta }{\theta +\mu }\right)}^{\theta }{\left(\frac{\mu }{\theta +\mu }\right)}^{x},$$

(4) $$ZINB\left(x;\pi ,\mu ,\theta \right)=\pi \delta_{0}\left(x\right)+\left(1-\pi \right)NB\left(x;\mu ,\theta \right).$$

where π, μ, and θ are the parameters of ZINB distribution, which represent the probability of dropout events, mean, and dispersion, respectively. DCA estimates three parameters by using an autoencoder framework; the formulation of the architecture is given below:

(5) $$\begin{array}{l} E=\text{Re}LU\left(XW_{E}\right),\\ B=\text{Re}LU\left(EW_{B}\right),\\ D=\text{Re}LU\left(BW_{\mu }\right),\\ M=diag\left(s_{j}\right)\exp\left(DW_{\mu }\right),\\ \Pi =sigmoid\left(DW_{\pi }\right),\\ \Theta =\exp\left(DW_{\theta }\right), \end{array}$$

where E, B, and D represent the encoder, bottleneck, and decoder layers, respectively. The loss function of DCA is the negative log of the ZINB likelihood:

(6) $$\hat{\Pi },\hat{\text{M}},\hat{\Theta }=argmin_{\Pi ,\text{M},\Theta }NLL_{ZINB}\left(X;\Pi ,\text{M},\Theta \right)+\lambda {||\Pi ||}_{F}^{2}.$$

where the *NLL*_*ZINB*_ function represents the negative log-likelihood of ZINB distribution.

### Graph Autoencoder Based on GATs

Graph autoencoder is a very powerful neural network architecture for unsupervised representation learning on graph-structured data. Compared with regular autoencoder, graph autoencoder applies graph neural networks in the encoder, which can better map the graph-structured data. In this work, we construct a graph autoencoder based on GAT to project the high-dimensional data to a low-dimensional latent space. GAT is a novel neural network architecture that extracts the features of the graph and preserves topological structure among cells.

Because the denoised data by DCA have the same dimension as the raw count, we select PCA to embed the gene expression matrix into an intermediate dimension. We select the first F principal components as the output matrix H of PCA. In this way, it can not only shorten the run time of the subsequent modeling but also enhance the performance of the KNN algorithm to build a more stable and accurate graph.

GAT aims to obtain a power expressive to transform the input feature H = {${\vec{h}}_{1}$,${\vec{h}}_{2}$,…,${\vec{h}}_{N}$} into higher-level feature *H*′ = {${\vec{h}}_{1}^{\prime }$,${\vec{h}}_{2}^{\prime }$,…,${\vec{h}}_{N}^{\prime }$}, ${\vec{h}}_{i}$∈$R^{F}$, and ${\vec{h}}_{i}^{\prime }$∈$R^{F^{\prime }}$. GAT learns the final output features of each node by using the information of their neighbor nodes:

(7) h→′i=∑j∈NiαijWh→j,

where α_*ij*_ represents the importance of node *j*’s features to node *i*, W is a shared weight matrix, and *j* ∈ *N*_*i*_, *N_i_* is some neighbor of node *i* in the KNN graph. The formula of α_*ij*_ is given below:

(8) $$\alpha_{ij}=softmax_{j}\left(e_{ij}\right)=\frac{exp\left(e_{ij}\right)}{\sum_{k\in N_{i}}exp\left(e_{ik}\right)},$$

where *e*_*ij*_ is the attention coefficient, it is defined as:

(9) $$e_{ij}=a\left(W{\vec{h}}_{i},W{\vec{h}}_{j}\right),$$

where the attention mechanism *a* is a single-layer feedforward neural network. To make coefficients *e*_*ij*_ (9) easily compare across different nodes, GAT applies softmax function to normalize them; we can obtain α_*ij*_ (8). GAT applies the LeakyReLU function as the activation function. After fully expanding out, the coefficients α_*ij*_ can be expressed as:

(10) $$\alpha_{ij}=\frac{exp\left(LeakyReLU\left({\vec{a}}^{T}\left[W{\vec{h}}_{i}||W{\vec{h}}_{j}\right]\right)\right)}{\sum_{k\in N_{i}}exp\left(LeakyReLU\left({\vec{a}}^{T}\left[W{\vec{h}}_{i}||W{\vec{h}}_{k}\right]\right)\right)},$$

where $\vec{a}\in R^{2F^{\prime }}$ is a weight vector, and || is the concatenation operation.

Our graph autoencoder has two inputs: compressed expression matrices H by PCA and adjacency matrices A. We apply GAT in the encoder. In our experiments, we encoder the inputs into two latent expressions, and then decode them into the reconstruct expression matrices *H*′ and adjacency matrices *A*′. The objective of the learning process is to minimize the reconstruction loss:

(11) $$L=\gamma {||H-H^{\prime }||}_{2}^{2}+\left(1-\gamma \right){||A-A^{\prime }||}_{2}^{2}.$$

where γ is a hyperparameter; we set it to be 0.6 in our experiments. It is a hyperparameter, which is used to balance the reconstruction loss of expression matrix and adjacent matrix. Since we mainly use the low-dimensional representation of adjacency matrix for subsequent dimensionality reduction and visualization, we pay more attention to the reconstruction loss of adjacency matrix, and then give more weight to the reconstruction loss of adjacency matrix.

### Convergence Analysis

SCDRHA consists of two core modules: DCA and graph autoencoder. How to train these two core modules is also a very important issue, and we give the setting of epochs when training them. Because we refer to the DCA in the process of noise reduction, we use the default value to train the DCA. For graph autoencoder, we first do pretraining, then global training; their epochs are set to 120 and 40, respectively. Because we find that when the number of epochs reaches this number, the value of the loss function of the graph autoencoder changes very little and tends to a more stable state; so, we have reason to think that the optimization objective tends to converge at this time.

## Results

### Datasets

To assess the performance of SCDRHA, we focus on relatively large datasets; five real scRNA-seq datasets with known cell types are selected. The basic information about five real datasets is summarized in Table 1, and below, we describe these datasets.

**TABLE 1:** Basic information about five real single-cell RNA sequencing (scRNA-seq) datasets.

Dataset	Cells	Genes	Clusters	Dropout rate (%)
10X PBMC	4,271	16,499	8	92.24
Mouse ES cell	2,717	24,046	4	65.76
Mouse bladder cell	2,746	19,079	16	94.87
Worm neuron cell	4,186	11,955	10	98.62
Zeisel	3,005	19,972	9	81.21

(i) The 10X PBMC (Zheng et al., 2017) dataset is provided by the 10X scRNA-seq platform, which is from a healthy human.^1^ (ii) The Mouse ES cell (Klein et al., 2015) dataset profiles the transcriptome of the heterogeneous onset of differentiation of mouse embryonic stem cells after Leukemia Inhibitory Factor (LIF) (Klein et al., 2015) withdrawal GSE65525. (iii) The Mouse bladder cell (Han et al., 2018) dataset is from the Mouse Cell Atlas project GSE108097. From the raw count matrix, we select about ∼2,700 cells from bladder tissue. (iv) The Worm neuron cell (Cao et al., 2017) dataset is profiled by single-cell combinatorial indexing RNA sequencing (sci-RNA-seq), which is from the nematode *Caenorhabditis elegans* at the L2 larval stage.^2^ (v) The Zeisel et al. (2015) dataset contains 3,005 cells, which are collected from the mouse cortex and hippocampus GSE60361.

### The Evaluation of SCDRHA in Dimensionality Reduction

In our experiments, four popular dimension reduction algorithms are used to compare with our algorithm SCDRHA in five real datasets. These four dimension reduction algorithms include two traditional algorithms (PCA and tSNE) and two novel algorithms for dimensionality reduction of scRNA-seq data (DCA and scGAE).

Firstly, we compare SCDRHA with PCA, t-SNE, and scGAE and use average silhouette value (Rousseeuw, 1987) to evaluate the performance of these methods. It is worth noting that we compress the data into 10 dimensions for comparison, except t-SNE, and do not modify the default parameters in the algorithm. Because the algorithm DCA compresses the data to 32 dimensions by default, it is not selected in this experiment.

As is shown in Table 2, only on the Mouse bladder cell dataset, t-distributed stochastic neighbor embedding (t-SNE) performs better than SCDRHA. On the other four datasets, the dimension reduction performance of SCDRHA is obviously better than other methods. The t-SNE is a non-linear dimension reduction algorithm widely used in single-cell dimension reduction and visualization; it can directly project high-dimensional data into two to three dimensions. The Mouse bladder cell dataset has 16 cell clusters; more clusters will distort the computation of average silhouette value.

**TABLE 2:** Average silhouette value under different datasets.

Dataset	PCA	t-SNE	scGAE	SCDRHA
10X PBMC	0.066	0.129	0.112	**0.469**
Mouse ES cell	0.019	0.346	0.337	**0.411**
Mouse bladder cell	0.019	**0.251**	0.032	0.193
Worm neuron cell	−0.143	0.042	–0.026	**0.315**
Zeisel	−0.112	0.113	0.193	**0.317**

*Bold values indicate the highest score in the row and the corresponding method has the best performance.*

In order to further test the dimension reduction performance of SCDRHA, we use the embedding expression of different dimensionality reduction methods for clustering analysis. Besides, Normalized Mutual Information (NMI) (Strehl and Ghosh, 2002) and Adjusted Rand index (ARI) (Rand, 1971) are used to evaluate the performance of clustering analysis. To make the results easily comparable across different methods, we employ K-means for clustering analysis and set the parameter K as the real number of clusters in each dataset.

As shown in Tables 3, 4, our experiments illustrate that SCDRHA is superior to other methods in all datasets. It is worth noting that SCDRHA overtakes t-SNE on the Mouse bladder cell dataset, which indicates that denoising single-cell data before dimension reduction can improve the performance of the subsequent analysis.

**TABLE 3:** Normalized Mutual Information (NMI) score under different datasets.

Dataset	PCA	t-SNE	DCA	scGAE	SCDRHA
10X PBMC	0.320	0.536	0.735	0.650	**0.793**
Mouse ES cell	0.518	0.594	0.856	0.787	**0.951**
Mouse bladder cell	0.522	0.673	0.648	0.664	**0.732**
Worm neuron cell	0.197	0.426	0.467	0.532	**0.752**
Zeisel	0.255	0.469	0.452	0.636	**0.727**

*Bold values indicate the highest score in the row and the corresponding method has the best performance.*

**TABLE 4:** ARI score under different datasets.

Dataset	PCA	t-SNE	DCA	scGAE	SCDRHA
10X PBMC	0.180	0.356	0.723	0.434	**0.781**
Mouse ES cell	0.224	0.594	0.852	0.771	**0.971**
Mouse bladder cell	0.226	0.413	0.529	0.442	**0.550**
Worm neuron cell	0.032	0.290	0.280	0.246	**0.674**
Zeisel	0.129	0.326	0.313	0.502	**0.627**

*Bold values indicate the highest score in the row and the corresponding method has the best performance.*

In a word, our experiments demonstrate that SCDRHA has batter performance in dimension reduction than that other existing methods.

### The Evaluation of SCDRHA in Noise Reduction

Since SCDRHA involves the module of noise reduction, we compare SCDRHA with other denoising methods including DCA, PRIME (Jeong and Liu, 2020), and DrImpute (Gong et al., 2018). These methods aim to impute dropout events in scRNA-seq data. At the same time, we also compare the denoised data with the original data.

**FIGURE 2:**
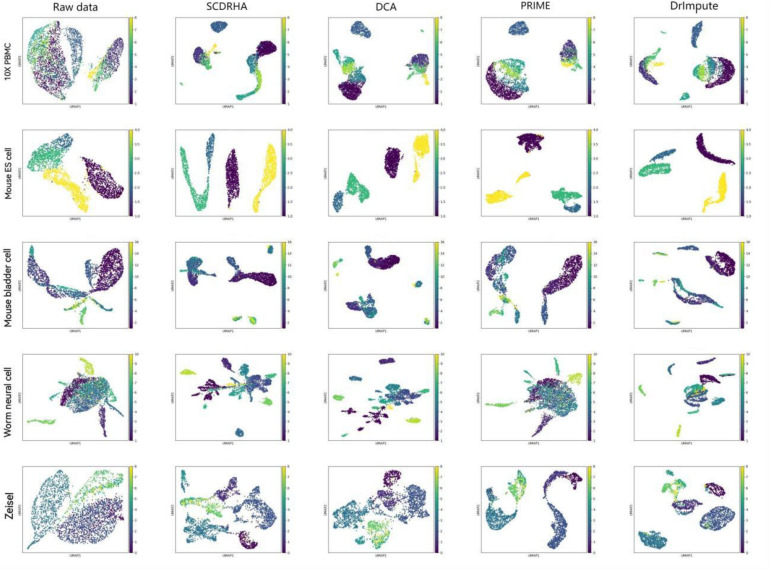
Cell visualization results for all single-cell RNA sequencing (scRNA-seq) datasets. The columns, from left to right, represent the raw data and the data visualization after noise reduction by SCDRHA, DCA, PRIME, and DrImpute. The rows, from left to right, represent visualizations of 10X PBMC, Mouse ES cell, Mouse bladder cell, Worm neuron cell, and Zeisel datasets.

Visualizing complex, high-dimensional scRNA-seq data in a way that is both easy to understand and faithful to the data is a meaningful task. To further evaluate the SCDRHA comprehensively, we employ UMAP to project the denoised data and the original data into two dimensions for cell visualization. Figure 2 shows the results of cell visualization for all five scRNA-seq datasets.

We can discover that SCDRHA can clearly divide different types of cells into different clusters. SCDRHA has a better performance on cell visualization than other methods. Comparing raw data with denoised data, we can find that SCDRHA remarkably improves the performance of data visualization. The results demonstrate that SCDRHA has a good ability for noise reduction.

To further evaluate the performance of noise reduction. We also apply K-means for clustering and use the NMI and ARI to assess their ability, thereby testing these methods indirectly. Before clustering analysis, we project the raw data and denoised data into the same dimensions by PCA.

The two metrics (NMI and ARI) of clustering performance are presented in Tables 5, 6. We observe that the clustering results of SCDRHA are better than other algorithms on the five selected datasets. In addition, denoising can significantly enhance the ability of clustering.

**TABLE 5:** NMI score under different datasets.

Dataset	Raw data	DrImpute	PRIME	DCA	SCDRHA
10X PBMC	0.320	0.716	0.682	0.735	**0.793**
Mouse ES cell	0.518	0.609	0.643	0.856	**0.951**
Mouse bladder cell	0.522	0.721	0.693	0.648	**0.732**
Worm neuron cell	0.197	0.665	0.376	0.467	**0.752**
Zeisel	0.255	0.605	0.574	0.452	**0.727**

*Bold values indicate the highest score in the row and the corresponding method has the best performance.*

**TABLE 6:** ARI score under different datasets.

Dataset	Raw data	DrImpute	PRIME	DCA	SCDRHA
10X PBMC	0.180	0.654	0.583	0.732	**0.781**
Mouse ES cell	0.224	0.474	0.497	0.852	**0.971**
Mouse bladder cell	0.226	0.477	0.463	0.529	**0.550**
Worm neuron cell	0.032	0.396	0.215	0.280	**0.674**
Zeisel	0.129	0.465	0.460	0.313	**0.627**

*Bold values indicate the highest score in the row and the corresponding method has the best performance.*

### Parameter Sensitivity Analysis

**FIGURE 3:**
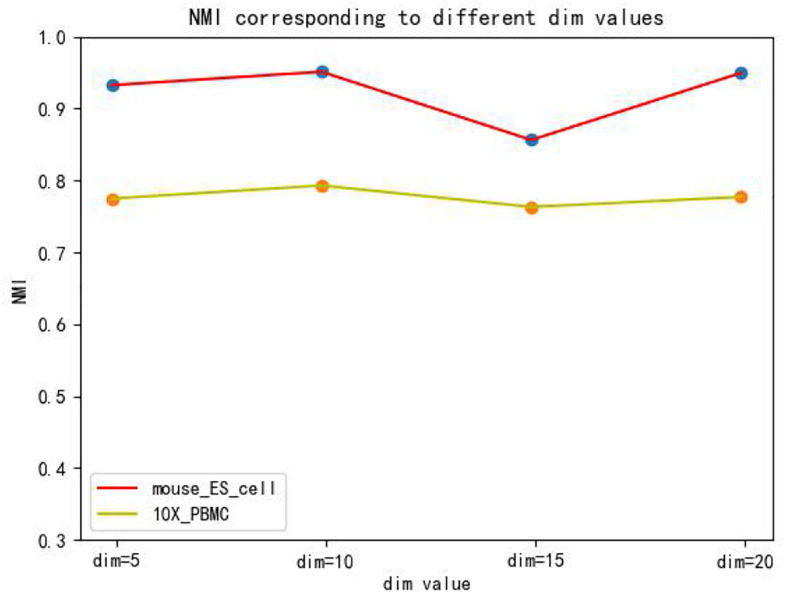
The influence of hidden layer nodes on SCDRHA under Normalized Mutual Information (NMI).

The hidden layer nodes of the graph autoencoder are a hyperparameter in SCDRHA, which directly determines the dimension of the final latent expression. To analyze the influence of the hidden layer nodes of graph autoencoder on SCDRHA, we select two datasets (Mouse ES cell and 10X PBMC) as the test datasets. The numbers of hidden layer nodes are set to 5, 10, 15, and 20, respectively. We use the latent space of different dimensions for clustering analysis. Three metrics are applied for analysis. The experimental results are summarized in Figure 3.

**FIGURE 4:**
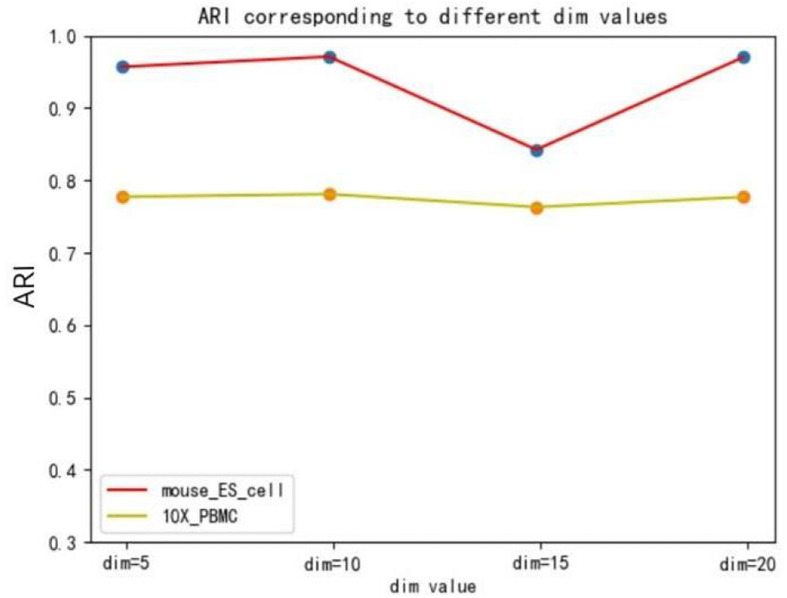
The influence of hidden layer nodes on SCDRHA under adjusted Rand index (ARI).

**FIGURE 5:**
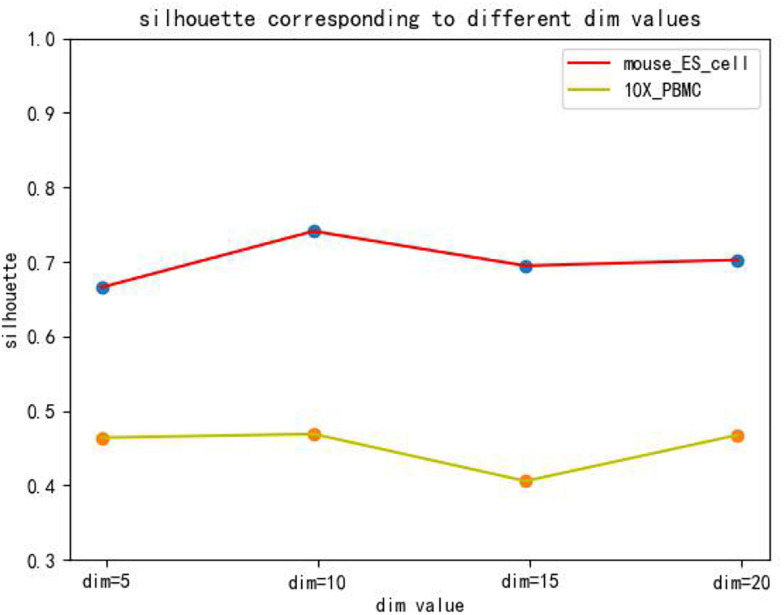
The influence of hidden layer nodes on SCDRHA under Silhouette.

Figures 3, 4, 5 show that different values of hidden layer nodes have a slight variation in the dimension reduction and clustering analysis, and when we selected the total number of nodes is 10, the performance under the three indexes is the best. Based on this analysis, the default parameter of the hidden layer nodes in graph autoencoder is set to 10.

### Implementation

The SCDRHA is implemented on HP Z840 workstation with 32GB RAM. SCDRHA consists of two portions: one is DCA and the other is graph autoencoder. We refer to the original code of DCA, which is constructed based on TensorFlow 1.15.0^3^ and implement DCA using SCANPY 1.7.1, a Python package. We refer to scGAE^4^ to build a graph autoencoder that is based on TensorFlow 2.4.1 and Python package spektral 0.6.1. Code and data used in this paper are available at https://github.com/WHY-17/SCDRHA.

### Software Package and Setting

When comparing with other methods, we followed the package and instructions provided by the author of each method. We basically use the default parameters of each package, and we used the following packages: (i) PRIME,^5^ (ii) DrImpute,^6^ (iii) DCA (see text footnote 3), and (iv) scGAE (see text footnote 4).

## Conclusion

Because of the high dimension of scRNA-seq, many dimension reduction methods have been proposed for scRNA-seq data in recent years. Nevertheless, these dimension reduction methods have some limitations in solving dropout events or maintaining local and global structure in the high-dimensional data. In conclusion, we propose SCDRHA, a scRNA-seq data dimensionality reduction algorithm based on a hierarchical autoencoder. scDeepCluster can learn a latent embedded representation that can denoise the data and preserve the topological structure. SCDRHA denoises the scRNA-seq data to obtain a more stable structure for the subsequent process. To obtain a low-dimension expression and retain the topological structure of single-cell data, we build a graph autoencoder based on GAT. Experimental results demonstrate that SCDRHA has better performance than existing state-of-the-art algorithms on dimension reduction and noise reduction in five real scRNA-seq datasets. Besides, SCDRHA can also dramatically enhance the performance of data visualization and cell clustering. With the rapid development of scRNA-seq technology, the data structure we get is more and more complex. Learning a more flexible and universal distribution to fit the data may be our future research direction.

## Data Availability Statement

Publicly available datasets were analyzed for this study. These can be found in the following links: 10X PBMC (https://support.10xgenomics.com/single-cell-gene-expression/), Mouse ES cell (https://www.ncbi.nlm.nih.gov/geo/query/acc.cgi?acc=GSE65525), Mouse bladder cell (https://www.ncbi.nlm.nih.gov/geo/query/acc.cgi?acc=GSE108097), Worm neuron cell (http://atlas.gs.washington.edu/worm-rna/docs/), and Zeisel (https://www.ncbi.nlm.nih.gov/geo/query/acc.cgi?acc=GSE60361).

## Author Contributions

NW, HW, and JZ constructed the original idea, designed the experiments, and wrote the manuscript. YS and CZ proofread the manuscript. All authors contributed to the article and approved the submitted version.

## Conflict of Interest

The authors declare that the research was conducted in the absence of any commercial or financial relationships that could be construed as a potential conflict of interest.

## Publisher’s Note

All claims expressed in this article are solely those of the authors and do not necessarily represent those of their affiliated organizations, or those of the publisher, the editors and the reviewers. Any product that may be evaluated in this article, or claim that may be made by its manufacturer, is not guaranteed or endorsed by the publisher.

## Footnotes

1
https://support.10xgenomics.com/single-cell-gene-expression/

2
http://atlas.gs.washington.edu/worm-rna/docs/

3
https://github.com/theislab/dca

4
https://github.com/ZixiangLuo1161/scGAE

5
https://github.com/hyundoo/PRIME

6
https://github.com/gongx030/DrImpute
